# Gut microbiota and glucometabolic alterations in response to recurrent partial sleep deprivation in normal-weight young individuals

**DOI:** 10.1016/j.molmet.2016.10.003

**Published:** 2016-10-24

**Authors:** Christian Benedict, Heike Vogel, Wenke Jonas, Anni Woting, Michael Blaut, Annette Schürmann, Jonathan Cedernaes

**Affiliations:** 1 Department of Neuroscience, Uppsala University, Uppsala, Sweden; 2 Department of Experimental Diabetology, German Institute of Human Nutrition Potsdam-Rehbruecke, Nuthetal, Germany; 3 German Center for Diabetes Research, Neuherberg, Germany; 4 Department of Gastrointestinal Microbiology, German Institute of Human Nutrition Potsdam-Rehbruecke, Arthur-Scheunert-Allee 114-116, 14558 Nuthetal, Germany

**Keywords:** Bacteroidetes, Firmicutes, Insulin resistance, Intestinal microbiome, Short-chain fatty acid, Sleep restriction, d2, day 2, F:B, Firmicutes:Bacteroidetes (ratio), HDL, high-density lipoprotein, HOMA-IR, homeostatic assessment model of insulin resistance, LDL, low-density lipoprotein, NS, normal sleep, OGTT, oral glucose tolerance test, OTU, Operational Taxonomic Units, PERMANOVA, permutational analysis of variance, PSD, partial sleep deprivation, SCFA, short-chain fatty acid, T2DM, type-2 diabetes mellitus

## Abstract

**Objective:**

Changes to the microbial community in the human gut have been proposed to promote metabolic disturbances that also occur after short periods of sleep loss (including insulin resistance). However, whether sleep loss affects the gut microbiota remains unknown.

**Methods:**

In a randomized within-subject crossover study utilizing a standardized in-lab protocol (with fixed meal times and exercise schedules), we studied nine normal-weight men at two occasions: after two nights of partial sleep deprivation (PSD; sleep opportunity 02:45–07:00 h), and after two nights of normal sleep (NS; sleep opportunity 22:30–07:00 h). Fecal samples were collected within 24 h before, and after two in-lab nights, of either NS or PSD. In addition, participants underwent an oral glucose tolerance test following each sleep intervention.

**Results:**

Microbiota composition analysis (V4 16S rRNA gene sequencing) revealed that after two days of PSD vs. after two days of NS, individuals exhibited an increased Firmicutes:Bacteroidetes ratio, higher abundances of the families Coriobacteriaceae and Erysipelotrichaceae, and lower abundance of Tenericutes (all P < 0.05) – previously all associated with metabolic perturbations in animal or human models. However, no PSD vs. NS effect on beta diversity or on fecal short-chain fatty acid concentrations was found. Fasting and postprandial insulin sensitivity decreased after PSD vs. NS (all P < 0.05).

**Discussion:**

Our findings demonstrate that short-term sleep loss induces subtle effects on human microbiota. To what extent the observed changes to the microbial community contribute to metabolic consequences of sleep loss warrants further investigations in larger and more prolonged sleep studies, to also assess how sleep loss impacts the microbiota in individuals who already are metabolically compromised.

## Highlights

• Possibly the first results of how short sleep impacts the human gut microbiota.
• Two nights of short sleep do not significantly impact beta diversity.
• The Firmicutes to Bacteroidetes ratio is significantly affected by sleep loss.
• Fecal short-chain fatty acid levels do not change depending on sleep duration.
• Increased insulin resistance after sleep loss is unrelated to alterations in the microbiota.

## 1 Introduction

The last two decades have revealed an increasingly important role of the gut microbiome for human health [1], [2], [3]. Differences in intestinal microbiota composition have been uncovered between healthy controls and those suffering from various diseases, including metabolic pathologies such as obesity, the metabolic syndrome and type-2 diabetes mellitus (T2DM) [4], [5], [6]. An important role of the gut microbiome for energy homeostasis is supported by studies showing that germfree mice – which lack a functional microbiome – are resistant to diet-induced obesity on a western diet [7]. Consistently, transplanting the microbiota of genetically obese ob/ob mice or obese humans to lean germfree mice increases the adiposity of the recipient mice [8], [9], indicating an increased capacity to harvest energy from gut nutrients in the obese state when identical nutrients are provided [8], [10]. Furthermore, similar transplantation of the microbiome from the obese but not normal-weight twin, to healthy germ-free mice, also promotes adiposity and perturbs glucose metabolism [9]. Providing evidence for the importance of a healthy microbiota, beneficial effects concerning insulin sensitivity have been observed in human trials of patients with metabolic syndrome who received the transplants from lean donors [4].

The gut microbiome regulates nutrient availability through complex interactions with dietary factors, and, consequently, the composition of the microbiota has been found to regulate the metabolic response to various such factors in humans [11], [12]. Diet is a key pathophysiological and treatment component for diseases such as T2DM [13], and diet has a major impact on the gut microbiome as it is the most important energy source for intestinal bacteria. Both acute [12], [14], [15], [16] and long-term [16], [17] changes in diet have been demonstrated to alter the abundance of bacterial species in the gut, as well as their functional capacity to process various nutrients, with such changes being detectable within days of dietary interventions in humans [15], [16].

Macronutrients in our diets enable bacteria in the large intestine to produce short-chain fatty acids (SCFAs), which include acetate, propionate, and butyrate. SCFAs can constitute about 2.2% of daily caloric intake [18], but also exert effects locally in the gut, as well as on hepatic glucose and lipid metabolism [19]. Butyrate and propionate have recently been demonstrated to play a role in intestinal gluconeogenesis, which has beneficial effects on glucose and energy homeostasis [20]. T2DM patients have been found to have a reduction in butyrate-producing bacterial species, and lower levels of butyrate biosynthesis when the microbiomes of T2DM patients are compared with those of non-diabetic controls [6]. Instead, obesity has been associated with an increase in total levels of SCFAs [10], and dietary factors can also alter these levels [19], [21]. Indeed, increased acetate production by gut bacteria has been found recently to promote insulin secretion, hyperphagia, and obesity [22], although there is also evidence that acetate is able to decrease appetite [23].

The risk of T2DM and obesity – metabolic pathologies that have been linked to dysregulated gut microbiotas – has been found to be increased in subjects suffering from chronic sleep loss [24], which has become increasingly common in modern stressful 24/7 lifestyle [25]. Merely curtailing sleep to half the recommended amount for a single night acutely impairs fasting insulin sensitivity [26]. When prolonged, sleep restriction can promote weight gain [27], possibly by altering energy expenditure as well as food choices and the behavioral response to especially hedonic food stimuli [24].

There is currently some evidence in both mice and humans that the gut microbiota exhibits a circadian rhythm [28], [29], [30] and, correspondingly, that this may be perturbed following circadian misalignment [28]. However, to date, there are no studies that have investigated the impact of insufficient sleep on the composition of the human gut microbiota. Studies are therefore lacking that assess whether important adverse metabolic changes that may increase the risk of T2DM and obesity, such as impaired insulin sensitivity, are associated with changes in the gut microbiome and associated SCFAs that could result from recurrent sleep loss. To this end, we conducted a study in which the impact of sleep loss on the human gut microbiota was assessed, in healthy, young normal-weight individuals.

## 2 Methods

### 2.1 Participants

Out of a total of 16 male individuals recruited for the present study, nine were able to provide fecal samples for both the baseline (see below) and second day measurement for both sessions, and were thus included for further analysis. All nine individuals had self-reported 24-hr sleep-wake within the recommended range as defined by the National Sleep Foundation (self-reported habitual sleep duration 7–9 h during nocturnal hours; sleep onset latency <30 min) and meal patterns (regular breakfast, lunch and dinner as main meals) as assessed by sleep diaries (data not shown). Participant data is shown in Table 1. Written and oral interviews of participants' current and prior physical and mental health were conducted by a medical doctor (J.C.) before participants were enrolled in the study, to ensure that participants were in general good health, did not use any medication or supplements, and that the participants did not suffer from or had been diagnosed with any psychiatric conditions, sleep disorders, or allergies. Participants reported not to suffer from any gastrointestinal disorder and did not experience regular or intermittent gastrointestinal discomfort. Furthermore, none of the participants had suffered from any diarrheal or infectious gastrointestinal disease in the last two years. All participants consumed a mixed normal diet, and none was a strict vegetarian. Furthermore, none of the participants reported having been born via caesarian section, and none of the participants had been treated with antibiotics during the previous twelve months.

During the morning screening visit, fasting glucose (<6.1 mmol/L) and normal blood count values were verified, followed by a 2-h oral glucose tolerance test (OGTT), which confirmed that all participants had normal glycemic control. Finally, about a week prior to the first session, participants underwent a night-long laboratory polysomnography, in order to habituate them to the experimental setting. All participants provided informed consent; the study was approved by the Regional Ethical Review Board in Uppsala (EPN 2014/242/1) and was conducted in accordance with the Helsinki Declaration.

### 2.2 Experimental procedure

A within-subject design was utilized for the current study: all participants took part in two separate experimental conditions (normal sleep, NS; vs. partial sleep deprivation, PSD) in which participants had an 8.5-h long sleep opportunity (22:30–07:00 h) in the normal sleep condition and a 4.25-h long sleep opportunity (2:45–07:00 h) in the PSD condition. Fecal samples were collected before and after 1) two nights of normal sleep and 2) after two nights of PSD. All experiments were conducted at our sleep laboratories at the Biomedical Center at Uppsala University, Sweden. Participants were randomly assigned which experimental condition they would start with (NS vs. PSD); this was counterbalanced for the total number of subjects included in the larger study set, of which results have previously been reported [31]. It should be noted, therefore, that in the present study, out of nine participants six started with their PSD condition, while three instead started with their normal sleep condition.

Participants maintained sleep diaries the week prior to admission to each of the two sessions and were instructed to try to go to bed around 22:00–24:00 h each night, and to get up after 7–9 h of sleep, around 6:00–8:00 h. The sleep diaries preceding each intervention did not reveal any significant differences for self-reported total sleep duration (P = 0.19, two-tailed). Participants were also instructed to maintain similar dietary and activity habits prior to each sleep intervention, including maintaining intake of breakfast, lunch and dinner around their previously documented regular hours. For each condition, participants came to the laboratory at 17:30 h on the arrival day (day 0). Participants were provided with a standardized dinner (33% of each participant's estimated daily calorie requirement), and were prepared for sleep (recorded by polysomnography). During the additional hours of wakefulness in the PSD condition (22:30–02:45 h), room lights were kept at <6 lux at eye-level.

As meal timing and nutrient composition can influence the gut microbiota [30], these were kept constant across the two study conditions. To further isolate the solitary impact of sleep on the microbiota – separate from the previously documented impact of e.g. high-fat diet [32] – participants were provided with a non-sugar sweetened, low-fat diet throughout the three meals, the exception being the oral glucose tolerance (OGTT) test on day 2; the OGTT was employed to test the participants' insulin sensitivity using the WHO-defined gold standard [33]. Participants were provided with yoghurt (Arla, 3 g fat/100 g) and natural muesli (ICA, 7 g fat/100 g) for breakfast; pasta bolognese for lunch (Findus, 2 g fat/100g); and a mix of potatoes, beef, rapeseed oil and onion (Findus, 5.5 g fat/100 g) for dinner. Both lunch and dinner were microwave-heated from frozen food packages within 15 minutes of each respective meal. On day 1, participants were provided with isocaloric meals (breakfast at 09:00 h, lunch at 13:00 h and dinner at 20:00 h; each providing 33% of each participant's estimated daily calorie requirement). On day 2, participants were first provided with a glucose solution (for the OGTT, at 08:30 h), and were then provided with a snack (400 kcal) around 11:00 h, followed by meal timing as on day 1, i.e. lunch at 13:00 h, and dinner at 20:00 h. Each meal had to be consumed in its entirety within 20 minutes of being served. Participants were allowed ad lib intake of regular water throughout the study except for during the OGTT in order to avoid dilution-induced differences in the rate of glucose uptake from the intestine.

To avoid a sedentary activity profile, participants were taken on three supervised walks at a standardized slow pace (walking pace ∼4 km/h; 10 min at 11:15 h, 30 min at 14:00 h and 20 min at 17:00 h) on day 1 and 2. Participants were otherwise confined to their rooms, where they could carry out activities at a sedentary level (e.g. reading, watching movies, playing board games) while being monitored by the experimenters.

On the morning of day 2, blood was collected through an indwelling catheter in the forearm, starting at around 08:30 h (fasting state), after which participants ingested the glucose solution for the OGTT (75 g glucose in 250 ml of water) within 60 s. After lying on their right side for five minutes (to ensure equal anatomical distribution of the glucose solution across all subjects; [34]), additional blood samples were collected every 30 min up to 2.5 h after ingestion of the glucose solution. Participants remained in a semi-recumbent position throughout the OGTT blood collection (∼08:15 h to ∼11:00 h).

Plasma lipids were obtained in the fasting state after another night of PSD or NS, this was followed by cognitive tests which have been reported elsewhere [31].

### 2.3 Collection of fecal samples

Sterile stool collection kits (Cat #K708, WA Products, UK) were handed out to the participants to collect fecal samples at home; these were also provided to the participants during their sessions in our sleep laboratory for continued fecal collection, which participants were encouraged to do whenever possible. Participants were provided with written and oral instructions on how to collect fecal samples before coming into the lab. These instructions were also repeated on the day that subjects were to collect their baseline samples, as well as when subjects arrived in the lab on each session. For baseline fecal samples, which were collected at home, participants were instructed to collect a sample as close as possible (within 24 h) to arriving at the sleep laboratory for each session. The participants were instructed to freeze the sample (to −18 °C or colder) in the provided sterile container, and to do so in a container filled with ice, enabling maintenance of sample integrity during the brief transport to the sleep laboratory, where samples were transferred to −80 °C freezer storage. Samples collected during the laboratory sessions were frozen at −80 °C as soon as possible; all samples were stored at −80 °C until further analyses. A total of 36 samples were utilized for the present analysis (nine subjects, two conditions, baseline and day 2 time points); these were aliquoted for subsequent analysis of microbial composition and SCFA content.

### 2.4 Microbial sequencing

For assessing the microbiota composition of the obtained fecal samples, nucleic isolation from fecal aliquots was carried out by Second Genome (San Francisco, CA, USA). The MoBio PowerMag^®^ Microbiome kit (Carlsbad, CA, USA) was utilized according to manufacturer guidelines, optimized for high-throughput processing. Extracted sample DNA content was quantified with the Qubit^®^ Quant-iT dsDNA High Sensitivity Kit (Invitrogen, Life Technologies, Grand Island, NY, USA). For subsequent library preparation, enrichment of bacterial 16S V4 rDNA region was achieved by amplifying DNA with the use of fusion primers, designed to target the surrounding conserved regions, incorporating Illumina (San Diego, CA, USA) adapters and indexing barcodes. PCR amplification was done with two different barcoded V4 fusion primers for each sample. Samples were then concentrated with a solid-phase reversible immobilization method for the purification of PCR products and qPCR quantified. Samples were finally pooled for 16S V4-enriched, amplified, barcoded sample sequencing, using MiSeq (Illumina, San Diego, CA), and run for 250 cycles with custom primers designed for paired-end sequencing. Samples were processed in a Good Laboratory Practices (GLP) compliant service laboratory, with Quality Management Systems for tracking of samples and data.

### 2.5 Analysis of short-chain fatty acids

Fecal SCFA levels were analyzed in duplicates and determined as previously described [35] with an HP 5890 series II gas chromatograph, but equipped with an HP-FFAP column. Helium served as carrier gas at a flow rate of 1 mL/min. SCFA concentrations were calculated using iso-butyric acid (12 mM) as internal standard.

### 2.6 Biochemical analyses

Plasma concentrations of glucose, triglycerides, as well as cholesterol levels (LDL, HDL, and total levels), were analyzed with an Architect C16000 chemistry analyzer (Abbott Laboratories, Chicago, USA). Serum insulin and cortisol were analyzed using commercially available ELISA kits (Cortisol Parameter Assay Kit, R&D Systems, Abington, UK).

### 2.7 Sleep assessment

To record sleep, Embla A10 recorders (Flaga hf, Reykjavik, Iceland) were employed, and EEG signals were derived from C3, C4, Fp1, Fp2 (referenced to the contralateral mastoid). An experienced scorer blinded to the study conditions adhered to standardized criteria for subsequent sleep analysis [36]. The polysomnographic analysis revealed that both on the first and second night, subjects slept 7 h:47min ± 13 min and 8 h:00 min ± 7 min, respectively, in the NS condition (i.e. over the minimum 7 h per night as recommended for adults by the US National Sleep Foundation). Instead, in the PSD condition, participants slept 3 h:59 min ± 4 min on the first night, and 4 h:3 min ± 3 min on the second night.

### 2.8 Statistical analysis

Second Genome's analysis software package was used for statistical analysis composition and diversity of the microbiome; this utilized permutational analysis of variance (PERMANOVA). Differential abundance of Operational Taxonomic Units (OTUs) was tested with a negative binomial noise model and an intrinsic Poisson process; this took into account both technical and biological variability between experimental conditions. For this analysis, DESeq was run with default settings and false discovery rate (FDR; q-values) was calculated with the Benjamini–Hochberg procedure. Wilcoxon signed rank test was performed for paired comparisons of microbial composition at the phylum or family level, whereas Student's t-test was used for other normally distributed parameters. Analyses of changes in the phyla, families, or OTUs of sequenced gut bacteria were analyzed for the eight most abundant features to minimize multiple comparisons. Repeated measures ANOVA was conducted to investigate the effects of PSD vs. NS on (**a**) glucose values (day 2), (**b**) insulin values (day 2), (**c**) baseline vs. day 2 differences across the sleep conditions for SCFAs, as well as for (**d**) the ratio of Firmicutes:Bacteroidetes. The factors *sleep* (PSD vs. NS) and *time* (baseline vs. day 2) were utilized for these ANOVAs; where applicable, normally distributed parameters were post-hoc analyzed using Student's t-test. Normal distribution of variables was assessed by the Shapiro–Wilk's test for normality; parameters not passing the normality test were log2-transformed before analysis. The Greenhouse–Geisser method was used to correct ANOVA analyses for sphericity deviations. Correlations were conducted using Pearson's correlation as all tested values were found to be normally distributed.

Two-sided P-values were utilized for the PERMANOVA, as we did not have any a priori hypothesis regarding the influence of sleep loss on beta diversity. One-sided P-values below 0.05 were considered significant for microbiota-derived parameters (such as the Firmicutes:Bacteroidetes ratio; [37], [38], [39], [40]) that have previously been associated with metabolic perturbations that also occur after acute sleep loss, including insulin resistance and dysregulation of lipid metabolism [34], [41]. Finally, as previous data have demonstrated increased insulin resistance following one or several nights of sleep loss, with increased postprandial but not baseline glucose levels [34], [41], one-sided P-values were used for postprandial markers of insulin resistance and for post-OGTT glucose values. Data are presented as means ± standard deviation (for all analyses of microbial composition) or standard error of the mean (S.E.M.; for all other analyses).

## 3 Results

### 3.1 Effects of partial sleep deprivation on microbial beta diversity

**Figure 1:**
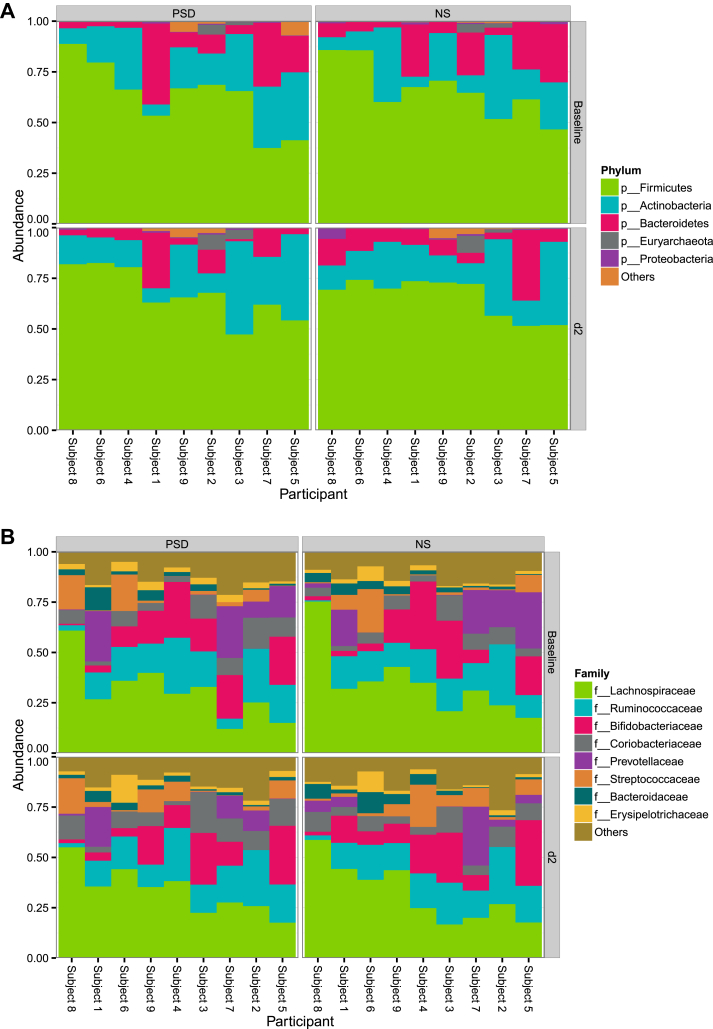

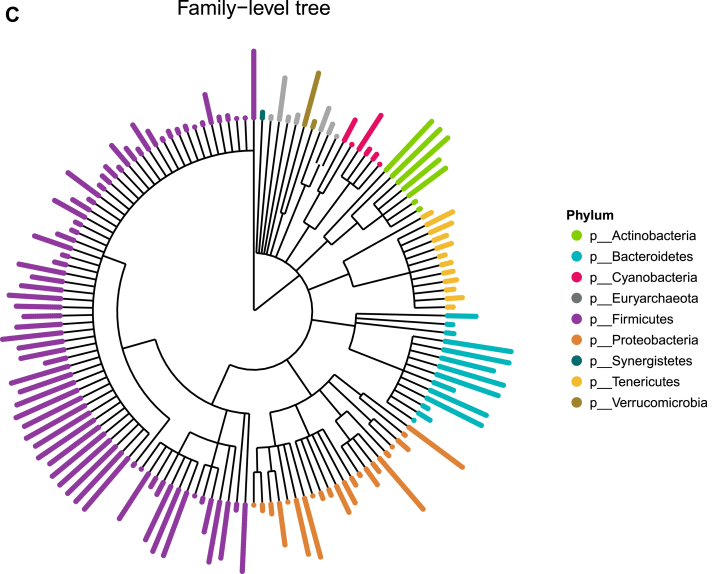
**(A)** High relative abundance of taxa within the phyla Firmicutes, Actinobacteria, and Bacteroidetes was observed across the fecal samples from the PSD (partial sleep deprivation) and normal sleep (NS) condition. Subjects are arbitrarily numbered and clustered along the y-axis based on bacterial phylum composition. **(B)** In an analysis across samples at the family level, high abundances were observed of Lachnospiraceae, Ruminococcaceae, Bifidobacteriaceae – and to lesser and more variable extent – of Coriobacteriaceae. **(C)** The microbiome sequencing analysis of samples obtained after sleep and PSD revealed 136 families; firmicutes was the most diverse phylum, containing the greatest number (69) of the classified families. d2, day 2.

**Figure 2:**
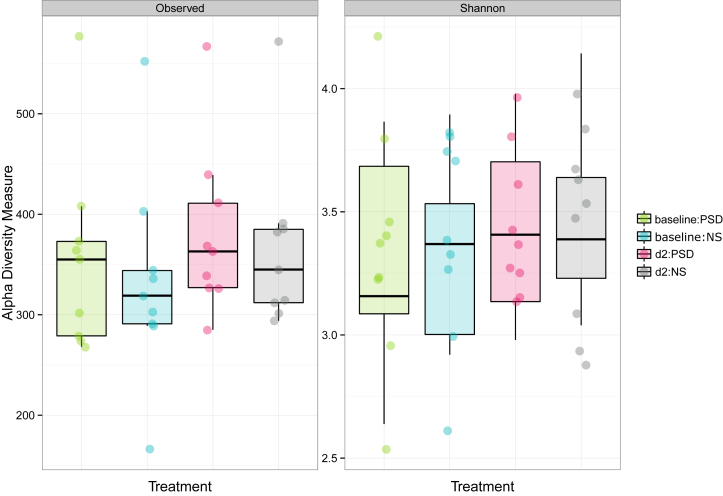
Within and across-subject variation tests for alpha diversity for both conditions (normal sleep, NS; vs. partial sleep deprivation, PSD) and time points (baseline vs. day 2 (d2) sample), using Observed and Shannon methods. See Supplementary Table 2 for statistical comparisons.

The number of filtered sequenced reads for microbes for the 36 analyzed fecal samples ranged from 52,465 to 237,072. This number was not significantly different between the two conditions (data not shown). The gut microbiome of the studied individuals exhibited a high relative abundance of taxa within the phyla Firmicutes, Actinobacteria, and Bacteroidetes (Figure 1A). Except for one subject, taxa from Lachnospiraceae, Ruminococcaceae, and Bifidobacteriaceae were also detected in the stool (Figure 1B). In total, the analysis revealed 14 phyla, 136 families, and a total of 546 detected species (Supplementary Table 1). Firmicutes was the most diverse phylum across samples, containing 69 of the 136 classified families (Figure 1C). However, it should be noted that out of this greater set of identified phyla, less than two percent belonged to phyla other than Firmicutes, Actinobacteria, Bacteroidetes, and Euryarchaeota (Figure 2A).

Testing for within and across-subject variation for both condition (NS vs. PSD) and time point (baseline vs. day 2), with alpha and beta diversity using Observed and Shannon methods, did not reveal any significant differences (Figure 2; all P > 0.05, two-sided; also Supplementary Table 2).

For each variable of interest, we utilized a PERMANOVA analysis using distance matrices to determine if the studied variables significantly contributed to the beta-diversity of the samples (Table 2). Neither BMI nor age significantly impacted beta diversity when only baseline fecal samples (from the first session; n = 9) were considered (age: P = 0.29, BMI: P = 0.677, both two-sided). Furthermore, neither sleep condition nor fecal collection day (time effect) were found to be significant drivers for beta diversity using weighted abundances or hierarchical clustering (baseline vs. day 2, P = 0.988; or day 2 samples for PSD vs. NS, P = 0.574, both two-sided; Supplementary Fig. 1A); in the clustering, samples were found to cluster primarily according to participant (Supplementary Fig. 1B). When we assessed each of the two studied conditions (PSD or NS) separately for baseline vs. day 2 differences, we also failed to find any impact on beta diversity over time (Table 2).

### 3.2 Effects of partial sleep deprivation on microbial composition at the phylum, family, and species level

In order to more closely examine the impact of sleep loss on the microbiome, we next examined changes in the microbiota composition at the phylum, family, and species level in response to our intervention. At baseline (i.e. before the sleep interventions were started), no within-subject differences in microbiota composition were noted, including for the beta diversity (P > 0.05 for all comparisons, two-sided). Following two nights of PSD vs. sleep, no significant changes were noted at the OTU level, whereas we observed a decrease in the Tenericutes phylum after PSD vs. NS (0.129 ±  0.241% vs. 0.284 ± 0.347%; P = 0.030, one-sided; Supplementary Fig. 2A; Table 3A), coupled with increased abundances of the families Coriobacteriaceae and Erysipelotrichaceae (9.61 ±  5.59% vs. 7.26 ±  3.30% and 3.37 ±  3.98% vs. 2.55 ±  2.97%, respectively; both P = 0.049, one-sided; Supplementary Fig. 2B; Table 3B).

Given that changes in the phyla Firmicutes and Bacteroidetes – and their ratio (F:B) – have been associated with obesity [40] and T2DM [39], we also specifically conducted targeted post-hoc analyses into how sleep loss impacted the relationship between these two phyla. A repeated measures ANOVA of the F:B ratio based on the proportional abundance of Firmicutes and Bacteroidetes revealed a trend for a main effect of sleep (P = 0.061, two-sided), but neither a time (P = 0.540, two-sided) nor interaction effect (P = 0.254, two-sided). Subsequent paired Student's t-tests indicated that the trend for the *sleep* effect was driven by an almost doubled F:B ratio after PSD vs. NS on day 2 (17.5 ±  13.7 vs. 9.1 ±  4.6, P = 0.04, one-sided), without any significant difference on baseline (13.1 ±  11.7 vs. 9.6 ±  7.2, P = 0.18, two-sided).

### 3.3 Changes in stool content of short-chained fatty acids following short sleep

We analyzed the levels of five different SCFAs – and total SCFA levels – in the samples obtained before and after two nights of NS and PSD. Across the studied SCFAs, we found trends for time effects for acetate, propionate, and for the total amount of fecal SCFAs (all P < 0.10, two-sided). Notably, while these effects descriptively seemed to be driven by decreasing levels of these SCFA parameters from baseline to d2 – especially in the PSD condition, we did not observe any main effects of *sleep* or any interaction effects, in the ANOVA (all P > 0.10, two-sided; Table 4).

### 3.4 Effects of partial sleep deprivation on glucometabolic parameters and lipid metabolism

**Figure 3:**
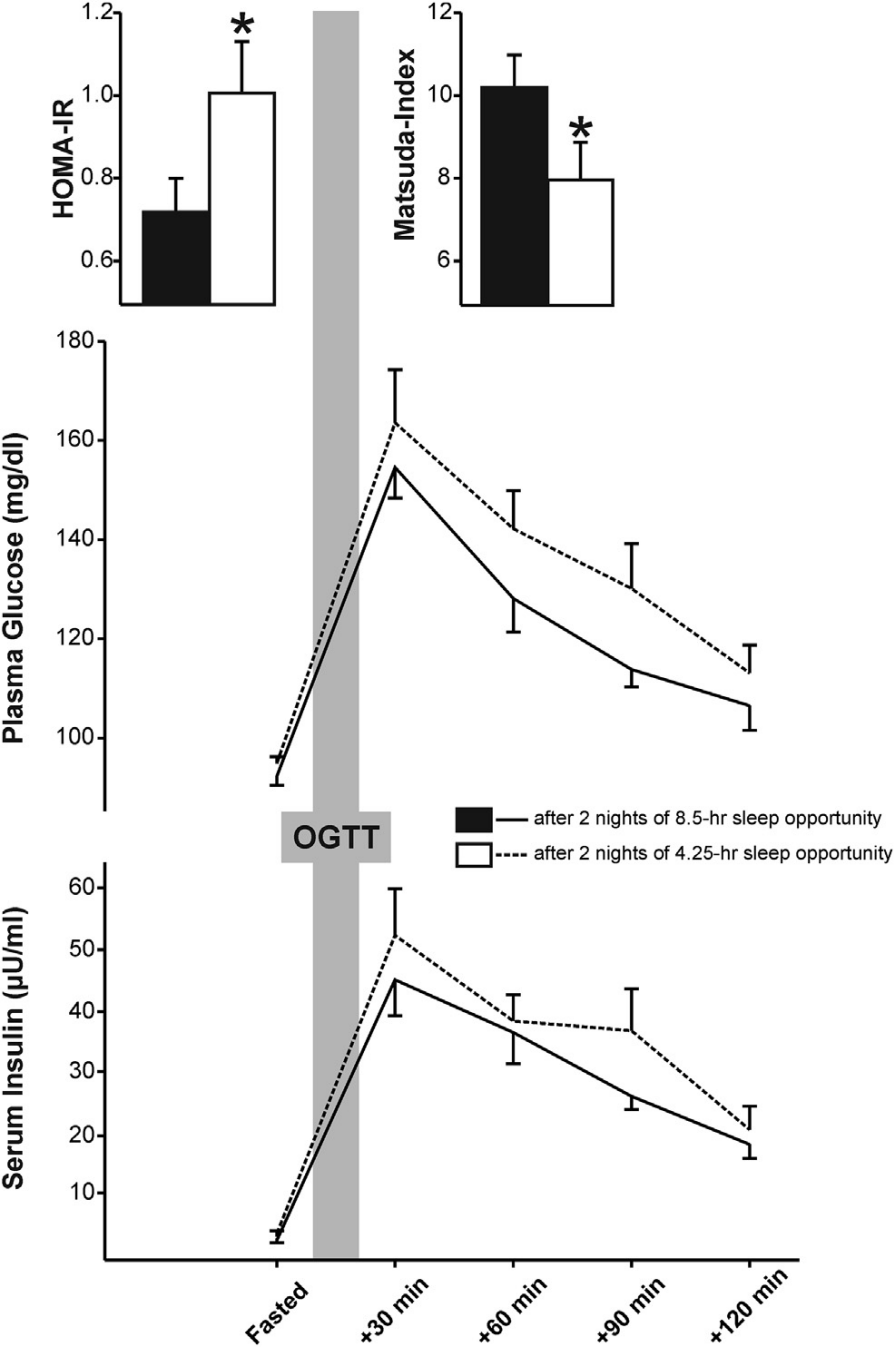
Glucometabolic values in response to normal sleep (black bar and solid lines) and partial sleep deprivation (white bar and dashed lines) for two consecutive nights. HOMA-IR and Matsuda index in the upper panel were obtained in the fasting state and from an oral glucose tolerance test (OGTT), respectively. Curves for plasma glucose (middle panel) and insulin (lower panel) were obtained from pre and post (up to 120 min) OGTT values. n = 9; *, P < 0.05.

After two days of restricted vs. normal sleep, participants exhibited almost 40% greater increased insulin resistance as assessed by the HOMA-IR index in the fasting state on the third day (1.01 ± 0.12 vs. 0.72 ± 0.08, P = 0.014, one-sided; Figure 3). Correspondingly, fasting insulin values were 34% higher following PSD than after NS (4.26 ± 0.48 vs. 3.17 ± 0.35 μU/mL, P = 0.006, one-sided), whereas glucose values were not significantly different at baseline (5.27 ± 0.07 vs. 5.12 ± 0.10 mmol/L; P = 0.14, one-sided). The subsequent OGTT demonstrated that two days of restricted sleep also decreased participants' postprandial insulin sensitivity by ∼22%, as assessed by the Matsuda index (8.0 ± 0.90 vs. 10.2 ± 0.78, P = 0.017, one-sided [42]; Figure 3). There were, however, no differences in the insulinogenic (0.78 ± 0.10 vs. 0.74 ± 0.14, P = 0.38, one-sided) or disposition index (5.97 ± 0.91 vs. 7.346 ± 1.57, P = 0.16, one-sided) after PSD vs. NS. Furthermore, separate repeated measures ANOVA for glucose and insulin values revealed a trend for a significant interaction effect for glucose but not insulin (F = 4.402, P = 0.069 for glucose; F = 3.177, P = 0.113 for insulin, both two-sided), and no main effect of sleep condition for either factor (F = 0.781, P = 0.546 for glucose; F = 0.284, P = 0.886 for insulin, both two-sided) (Figure 3). Instead, significant time effects were noted for both factors (F = 32.011, P = 0.000 for glucose; F = 89.499, P = 0.000 for insulin, both two-sided).

Given that the microbiota and microbiota-derived products such as SCFAs have been shown to exert effects on metabolism, specifically on insulin sensitivity, we also investigated whether beta diversity on day 2 was associated with the decreased insulin sensitivity observed on the same day following sleep loss, but we did not find any significant effect (PERMANOVA P = 0.121 and P = 0.235 for effects related to the HOMA-IR and Matsuda index, respectively, both two-sided).

As our results indicated a shift in the F:B ratio, we investigated whether this was related to changes in HOMA-IR or the Matsuda index within each sleep condition. We found that the F:B ratio at baseline in the NS condition was significantly positively correlated with the second day HOMA-IR (baseline: r = 0.79, P = 0.006; d2: r = 0.16, P = 0.39, both one-sided) as well as with the OGTT-derived Matsuda index (baseline: r = −0.75, P = 0.011; d2, r = −0.33, P = 0.20, both one-sided). In contrast, correlations with the baseline fecal sample were observed only for the HOMA-IR index in the PSD condition (HOMA-IR, baseline: r = 0.62, P = 0.038; d2: r = 0.25, P = 0.26; Matsuda index, baseline: r = −0.36, P = 0.17; d2: r = −0.03, P = 0.47, all one-sided).

We also assessed changes in plasma levels of LDL, HDL, total cholesterol, as well as triglyceride levels but did not find any significant differences between PSD and NS in our limited number of subjects (all P > 0.05, two-sided; Table 5), suggesting that the observed changes to Coriobacteriaceae and Erysipelotrichaceae – previously linked to obesity and lipid, primarily cholesterol, metabolism [43], [44], [45] – did not acutely impact lipid metabolism.

## 4 Discussion

Given that sleep loss negatively impacts glucose metabolism and that the gut microbiome can respond rapidly to exogenous factors such as diet, we carried out the first investigation into whether recurrent restricted sleep duration alters the human gut microbiota and whether these changes are associated with altered insulin sensitivity as a result of sleep loss. Two days of short vs. normal sleep was found to reduce insulin sensitivity without any associated changes in gut species diversity, which has previously been found to be altered in obese or T2DM patients vs. healthy normal-weight subjects.

We observed an increased Firmicutes:Bacteroidetes ratio following two nights of restricted sleep vs. normal sleep. An increase in this ratio in some [40], but not all [10] studies, has been found to be associated with obesity in humans as well as in genetically or diet-induced obese mice [40], [46], [47], and weight loss can increase the already lower abundance of Bacteroidetes in obese subjects [40]. Complementary to our findings, increased abundance of Firmicutes and reduced abundance of Bacteroidetes in the gut microbiota have been observed in mice subjected to sleep fragmentation for 4 weeks [48]. Such changes may promote increased energy uptake from the gut [8], [49], and could therefore constitute a possible mechanism through which chronic sleep loss can increase the risk of weight gain. However, we did not find any significance between sleep-loss induced changes in insulin sensitivity and the Firmicutes:Bacteroidetes ratio. This suggests that changes in the proportions of the dominant phyla in the gut may not represent – at least in the short-term – a central mechanism through which one or several nights of curtailed sleep reduces insulin sensitivity in humans. Other factors may be more causally involved, including changes in peripheral tissues' circadian clocks [34], together with associated and independent perturbations in metabolic pathways regulating peripheral glucose metabolism, insulin signaling, or proper insulin secretion from the pancreas [50], [51], [52].

Notably, we observed increased levels of the families Coriobacteriaceae and Erysipelotrichaceae (phylum Actinobacteria and Firmicutes, respectively), with a lower abundance of the phylum Tenericutes, following two nights of PSD compared with two nights of normal sleep. These increased levels have been associated previously with changes in hepatic and lipid metabolism in humans and various rodent species [44], [45], [53], [54], [55]. Notably, Tenericutes has been demonstrated to decrease following diet-induced obesity in mice [47], and among families of the phylum Firmicutes, specifically Erysipelotrichaceae has instead been found to increase several fold in mice exposed to both a high-fat or Western diet [56]. Consistent with these animal findings, smaller studies in humans have found that morbidly obese subjects harbor higher proportions of Coriobacteriaceae and Erysipelotrichaceae [43] and that levels of the latter positively correlate with changes in liver fat following a 2-month long dietary choline manipulation [44]. Given that we did not observe any changes to systemic markers of lipid and cholesterol metabolism, it remains to be determined whether the changes in these families of gut bacteria are more accentuated in chronic sleep loss conditions and, if so, whether these changes over longer time periods are associated with sleep loss-induced impaired metabolic integrity, such as increased risk of weight gain [57] and disrupted lipid metabolism [58], [59].

To determine whether our sleep loss paradigm functionally affected the intestinal microbiome, we measured levels of fecal SCFAs. These are produced by colonic bacteria and serve important metabolic roles after being readily absorbed through the large intestinal wall [19]. Both animal and human studies indicate that cholesterol synthesis and other liver functions can be influenced by gut-derived SCFAs, even over short time scales [60], [61], [62], [63]. Bacteria producing SCFAs are less abundant in obesity and T2DM [6], but, strikingly, some studies found higher fecal SCFA concentrations in obese subjects [10], [64]. Evidence suggests that supplementation with these molecules may impact insulin sensitivity and energy expenditure in mice and humans [65], [66]. High-fat diet feeding and/or obesity in mice and humans also alters the production of SCFAs [10], [21], and SCFAs have been found either to be permissive or protective against detrimental effects of diet-induced obesity on metabolism in mice [22], [65], [67]. As they readily cross the blood–brain barrier [22], SCFAs seem to be able to modulate behaviors such as appetite, even in humans [66], although the directionality remains to be fully determined [22], [23]. Herein, we failed to find any impact on SCFAs as a result of our sleep intervention, apart for statistical trends for time effects on acetate, propionate, and total fecal SCFAs levels. The lack of observed changes in fecal SCFA levels in our study suggests that changes in SCFAs are not causally involved in detrimental effects on metabolism that result from acute sleep loss. Nevertheless, whereas previous evidence has indicated that circadian disruption may impair the intestinal barrier [68], at present it is not known if pro-inflammatory effects of sleep loss can increase intestinal permeability in humans, and thus alter uptake and plasma levels of SCFAs, which ultimately will determine the metabolic and behavioral effects of SCFAs.

Apart from ingested nutrients and other dietary factors, the gut microbiota interacts with several local intestinal factors, including the local immune system and bile acids. Of note, bile acid components have been found to exhibit diurnal fluctuations in humans, and disrupting sleep or circadian rhythms has been demonstrated to impact levels of bile acids and their secretion in mouse models [69], [70], [71]. Such changes in intestinal bile acid levels, through altered production, secretion, and/or uptake (via bile acid transporters) [72], together with bidirectional effects of altered intestinal permeability also may represent mechanistic pathways through which changes in sleep duration or the sleep-wake cycle are able to impact the human gut microbiota and its functional relevance for metabolic integrity.

## 5 Limitations and perspectives

We utilized a standardized in-lab protocol (including for factors such as meal times and activity schedule) in an attempt to isolate the effect of sleep loss on the gut microbiome in normal-weight, healthy subjects. Obesity and associated metabolic perturbations primarily have been reflected by altered diversity of the microbiota [73], [74], but, in our limited number of individuals, we failed to observe a similar impact from our acute sleep restriction paradigm. It is, therefore, of future interest to investigate whether obesity is able to exacerbate any negative impact that sleep loss has on the gut flora and to what extent such effects may also be modulated by e.g. high- or high-sugar diets, which are known to impact not only glucose metabolism but also the gut microbiome [75].

A within-subject design was utilized to minimize bias due to between-subject comparisons. However, since only a subsample of the participants was able to provide fecal samples, we cannot rule out that the lack of counterbalancing in the order of experimental conditions may have influenced the present results. It should be kept in mind, however, that at baseline, no within-subject differences in the fecal microbiota composition were found, indicating that participants followed our instructions to maintain their individual dietary and activity habits (including bedtimes) prior to each sleep intervention. Nonetheless, longer studies that sample feces after an initial controlled baseline period are required to fully clarify how diet and exercise practices may alter the impact of sleep loss on the gut microbiota in humans.

Notably, whereas we studied the effects on the microbiota of restricting sleep in the first part of the night, possible circadian effects on the gut microbiota may be impacted by sleep timing, as indicated by previous studies [28], [29], [30], [68], but which remain to be fully explored. Longer sleep intervention studies that also include recovery sleep periods may also reveal to what extent sleep loss-induced effects on the gut microbiota are reversed when sleep-wake homeostasis is restored, similar to what has recently been observed in the context of sleep loss-induced metabolic perturbations [76], [77].

An important aspect is that the development of obesity and type 2 diabetes occurs over the span of years or decades. In this context, it should be emphasized that we only studied participants under two days and nights of controlled laboratory conditions. Thus, more long-term studies are warranted to examine the relationship between changes in the gut microbiota and curtailed sleep as a driving factor. As such, it will be important to examine both the persistence of acute changes in the gut microbiota, their functional relevance, and whether such changes also are observed in cross-sectional studies that examine the relationship between the gut microbiota and sleep. Even so, dietary-induced acute changes of the gut microbiota – observed in previous human studies [15], [16] – highlight how the gut microbiota interacts with dietary factors also over shorter timescales. In the present study, we employed an OGTT on the second day, revealing impaired insulin sensitivity in our participants when subjected to sleep loss. The exact timescale on which such acute dietary interventions (of different nutrient composition) occur remain undetermined. This is especially relevant in the setting of sleep loss or circadian misalignment, as such conditions may also impact gut motility [78] and thus the circadian timing of nutrient availability to the gut microbiota, as well as bowel emptying; the latter being of relevance when considering fecal sampling across the sleep-wake cycle.

Of note, a recent meta-analysis has thrown into question whether there is a specific gut microbiome signature of obesity [79]. As this relationship has been extensively explored for over a decade, this suggests that the sleep-microbiome connection will require numerous larger studies, as well as cross-sectional and longitudinal studies, to fully determine the role of the gut microbiome in relation to sleep and associated metabolic processes.

Finally, similar to what the last decade or so has revealed through the use of genetic profiling, microbiome profiling may also help understand inter-individual differences in the behavioral and physiological response to sleep loss. Changes in the gut microbiota can be caused by a range of factors (e.g. rapid dietary changes, antibiotics, fecal microbiome transplants). Thus, the gut microbiome represents a potential new field of research for developing tools that can be tailored for individuals who are at risk of developing negative metabolic health consequences due to chronic sleep loss or, as in shift workers, due to chronic circadian misalignment.

## 6 Conclusions

Results from our within-subject crossover study suggest that overall changes in the diversity of the gut microbiota may not be a driving factor behind acutely impaired glucose metabolism in response to recurrent partial sleep deprivation. Given our small sample size that only involved healthy young men, larger and more long-term studies are required to investigate to what extent these findings persist over longer time periods and whether these are observed in females, older or diseased patients and in other sleep restriction paradigms. Nevertheless, our study is the first to provide evidence for sleep deprivation-induced changes in microbial families of bacterial gut species, which have previously been linked to metabolic pathologies.

## Funding

Work from the authors' laboratory is supported by AFA Försäkring (CB); the Bissen Brainwalk Foundation (JC); Erik, Karin and Gösta Selanders Foundation (JC); Fredrik och Ingrid Thurings Foundation (JC), the Lars Hiertas Minne Foundation (JC); Novo Nordisk Foundation (CB); the Tore Nilson Foundation (JC); the Swedish Society for Medical Research (JC); the Swedish Society for Medicine (JC); the Swedish Brain Foundation (JC, CB); the Swedish Research Council (CB, JC); and the Åke Wiberg Foundation (JC).

## Duality of interest

The authors are unaware of any affiliation, funding, or financial holdings that might be perceived as affecting the objectivity of this manuscript. The authors declare that there is no duality of interest associated with this manuscript.

## Contribution statement

JC and CB designed the study; JC and CB wrote the protocol; JC collected the data; JC and CB conducted the analyses following initial bioinformatical analyses from Second Genome. Second Genome did not have any role in study design or interpretation of the results; nor in the writing process. All authors interpreted the data; and all authors contributed to writing. All authors have approved the final manuscript. We would like to thank the participants for their essential contribution. We would also like to thank Emil K. Axelsson, Jan-Erik Broman, Sara Hassanzadeh, Lauri Lampola, Lisanne Liethof, Olof Ros, Filip Sand and Adine Yeganeh for their help in conducting the study.

## Figures and Tables

**Table 1:** Baseline data for the study participants (n = 9).

Parameter	Mean ± S.E.M.
Age	23.3 ± 0.6 years
Body mass index	23.1 ± 0.6 kg/m^2^
Waist circumference	79.1 ± 2.0 cm
Hip circumference	98.2 ± 1.6 cm
Waist:hip ratio	0.80 ± 0.02

**Table 2:** PERMANOVA analysis results to determine if the investigated contrasts or parameters contributed to beta diversity of the samples. Neither body mass index (BMI) nor age significantly impacted beta diversity when only baseline fecal samples (from the first session; n = 9) were considered (see main text). Baseline, baseline sample; d2, day 2; HOMA-IR, Homeostatic model assessment of insulin resistance; NS, normal sleep; OGTT, oral glucose tolerance test; PSD, partial sleep deprivation. All P-values are two-sided.

Variable	P value	Sample count
Time point	0.991	18, 18
Age	0.001	36
BMI	0.004	36


Paired tests	P value	Sample count
d2 PSD_vs. d2 NS	0.581	9, 9
Baseline NS vs. d2 NS	0.455	9, 9
Baseline PSD vs. d2 PSD	0.632	9, 9
HOMA-IR d2	0.121	18
OGTT Matsuda index	0.235	18

**Table 3:** Wilcoxon signed rank test on the eight most abundant phyla **(A)** and families **(B)** in fecal samples collected after two nights (d2) of either partial sleep deprivation (PSD) or normal sleep (NS). Percent relative abundance means are shown and values are shown as mean ± SD. One-sided P values are shown (significance is displayed in bold font). V, Wilcoxon test statistic value.

Phylum	NS d2	PSD d2	V	P value
Firmicutes	65.8 ± 9.6	67.2 ± 12.5	28	0.277
Actinobacteria	20.2 ± 11.6	21.7 ± 14.2	28	0.277
Bacteroidetes	10.7 ± 9.53	8.2 ± 8.4	13	0.143
Euryarchaeota	1.12 ± 2.76	1.34 ± 2.74	20	0.407
Verrucomicrobia	0.813 ± 1.75	0.839 ± 1.49	17	0.277
Proteobacteria	0.992 ± 1.72	0.498 ± 0.42	15	0.204
Tenericutes	0.284 ± 0.347	0.129 ± 0.241	4	**0.030**
Cyanobacteria	0.0549 ± 0.15	0.11 ± 0.15	34	0.097


Family	NS d2	PSD d2	V	P value
Lachnospiraceae	32.4 ± 14.6	33.5 ± 11.6	24	0.453
Ruminococcaceae	16 ± 7.02	16.4 ± 7.84	20	0.407
Bifidobacteriaceae	12.9 ± 10.8	11.9 ± 10.6	18	0.318
Coriobacteriaceae	7.26 ± 3.3	9.61 ± 5.59	37	**0.049**
Streptococcaceae	6.13 ± 6.36	5.96 ± 6.19	22	0.5
Prevotellaceae	5.29 ± 9.28	4.73 ± 7.26	22	0.5
Bacteroidaceae	3.84 ± 3.43	2.28 ± 1.55	12	0.118
Erysipelotrichaceae	2.55 ± 2.97	3.37 ± 3.98	37	**0.049**

**Table 4:** Fecal levels of individual and total short-chained fatty acids in the normal sleep (NS) and partial sleep deprivation (PSD) condition, at baseline and after two days of each intervention (d2). Statistical trends (0.10 > P > 0.05; two-sided values) in the ANOVA are indicated in italic bold. Values shown as µmol/g Dry mass (DM) and as means ± S.E.M.

Parameter	NS baseline	NS d2	PSD baseline	PSD d2	Sleep effect	Time effect	Interaction effect
Acetate (C2) [µmol/g DM]	297.6 ± 45.6	290.3 ± 37.5	287.4 ± 44.4	203.2 ± 29.6	P = 0.375	***P = 0.080***	P = 0.261
Propionate (C3) [µmol/g DM]	77.6 ± 11.2	68.9 ± 8.0	70.2 ± 11.2	60.7 ± 10.4	P = 0.545	***P = 0.077***	P = 0.967
Butyrate (C4) [µmol/g DM]	67.0 ± 16.4	60.6 ± 7.3	64.0 ± 9.5	52.45 ± 25.17	P = 0.872	P = 0.454	P = 0.418
Valerate (C5) [µmol/g DM]	*7.6 ± 2.1*	*7.8 ± 1.2*	*7.3 ± 1.2*	*6.1 ± 0.7*	P = 0.376	P = 0.172	P = 0.220
Iso-valerate (iC5) [µmol/g DM]	9.9 ± 2.1	11.3 ± 1.3	8.9 ± 1.0	10.4 ± 1.4	P = 0.690	P = 0.168	P = 0.816
Total SCFAs [µmol/g DM]	459.8 ± 70.5	438.9 ± 51.0	437.8 ± 56.5	332.8 ± 43.7	P = 0.445	***P = 0.084***	P = 0.374

**Table 5:** Plasma levels of triglycerides, and of HDL, LDL, and total cholesterol following three nights of normal sleep (NS) or partial sleep deprivation (PSD). P values derive from paired Student's t-tests, (n = 9). Values shown as mean ± S.E.M.

Parameter	NS	PSD	P value
Triglycerides, mmol/l	0.84 ± 0.13	0.81 ± 0.12	0.37
Cholesterol, mmol/l	3.96 ± 0.19	3.79 ± 0.24	0.36
LDL, mmol/l	2.25 ± 0.14	2.03 ± 0.16	0.14
HDL, mmol/l	1.23 ± 0.13	1.26 ± 0.13	0.53
HDL/LDL	1.97 ± 0.22	1.79 ± 0.23	0.24

## References

[1] Broussard J.L., Devkota S. (2016). The changing microbial landscape of Western society: diet, dwellings and discordance. Molecular Metabolism.

[2] Cani P.D., Knauf C. (2016). How gut microbes talk to organs: the role of endocrine and nervous routes. Molecular Metabolism.

[3] Ussar S., Fujisaka S., Kahn C.R. (2016). Interactions between host genetics and gut microbiome in diabetes and metabolic syndrome. Molecular Metabolism.

[4] Vrieze A., Van Nood E., Holleman F., Salojarvi J., Kootte R.S., Bartelsman J.F. (2012). Transfer of intestinal microbiota from lean donors increases insulin sensitivity in individuals with metabolic syndrome. Gastroenterology.

[5] Hartstra A.V., Bouter K.E., Backhed F., Nieuwdorp M. (2015). Insights into the role of the microbiome in obesity and type 2 diabetes. Diabetes Care.

[6] Qin J., Li Y., Cai Z., Li S., Zhu J., Zhang F. (2012). A metagenome-wide association study of gut microbiota in type 2 diabetes. Nature.

[7] Backhed F., Manchester J.K., Semenkovich C.F., Gordon J.I. (2007). Mechanisms underlying the resistance to diet-induced obesity in germ-free mice. Proceedings of the National Academy of Sciences of the United States of America.

[8] Turnbaugh P.J., Ley R.E., Mahowald M.A., Magrini V., Mardis E.R., Gordon J.I. (2006). An obesity-associated gut microbiome with increased capacity for energy harvest. Nature.

[9] Ridaura V.K., Faith J.J., Rey F.E., Cheng J., Duncan A.E., Kau A.L. (2013). Gut microbiota from twins discordant for obesity modulate metabolism in mice. Science.

[10] Schwiertz A., Taras D., Schafer K., Beijer S., Bos N.A., Donus C. (2010). Microbiota and SCFA in lean and overweight healthy subjects. Obesity (Silver Spring).

[11] Kovatcheva-Datchary P., Nilsson A., Akrami R., Lee Y.S., De Vadder F., Arora T. (2015). Dietary fiber-induced improvement in glucose metabolism is associated with increased abundance of Prevotella. Cell Metabolism.

[12] Zeevi D., Korem T., Zmora N., Israeli D., Rothschild D., Weinberger A. (2015). Personalized nutrition by prediction of glycemic responses. Cell.

[13] Chan J.C., Malik V., Jia W., Kadowaki T., Yajnik C.S., Yoon K.H. (2009). Diabetes in Asia: epidemiology, risk factors, and pathophysiology. JAMA.

[14] Turnbaugh P.J., Ridaura V.K., Faith J.J., Rey F.E., Knight R., Gordon J.I. (2009). The effect of diet on the human gut microbiome: a metagenomic analysis in humanized gnotobiotic mice. Science Translational Medicine.

[15] David L.A., Maurice C.F., Carmody R.N., Gootenberg D.B., Button J.E., Wolfe B.E. (2014). Diet rapidly and reproducibly alters the human gut microbiome. Nature.

[16] Wu G.D., Chen J., Hoffmann C., Bittinger K., Chen Y.Y., Keilbaugh S.A. (2011). Linking long-term dietary patterns with gut microbial enterotypes. Science.

[17] Muegge B.D., Kuczynski J., Knights D., Clemente J.C., Gonzalez A., Fontana L. (2011). Diet drives convergence in gut microbiome functions across mammalian phylogeny and within humans. Science.

[18] Blaut M. (2015). Gut microbiota and energy balance: role in obesity. Proceedings of the Nutrition Society.

[19] Rios-Covian D., Ruas-Madiedo P., Margolles A., Gueimonde M., de Los Reyes-Gavilan C.G., Salazar N. (2016). Intestinal short chain fatty acids and their link with diet and human health. Frontiers in Microbiology.

[20] De Vadder F., Kovatcheva-Datchary P., Goncalves D., Vinera J., Zitoun C., Duchampt A. (2014). Microbiota-generated metabolites promote metabolic benefits via gut-brain neural circuits. Cell.

[21] Murphy E.F., Cotter P.D., Healy S., Marques T.M., O'Sullivan O., Fouhy F. (2010). Composition and energy harvesting capacity of the gut microbiota: relationship to diet, obesity and time in mouse models. Gut.

[22] Perry R.J., Peng L., Barry N.A., Cline G.W., Zhang D., Cardone R.L. (2016). Acetate mediates a microbiome-brain-beta-cell axis to promote metabolic syndrome. Nature.

[23] Frost G., Sleeth M.L., Sahuri-Arisoylu M., Lizarbe B., Cerdan S., Brody L. (2014). The short-chain fatty acid acetate reduces appetite via a central homeostatic mechanism. Nature Communications.

[24] Cedernaes J., Schioth H.B., Benedict C. (2015). Determinants of shortened, disrupted, and mistimed sleep and associated metabolic health consequences in healthy humans. Diabetes.

[25] Ford E.S., Cunningham T.J., Croft J.B. (2015). Trends in self-reported sleep duration among US Adults from 1985 to 2012. Sleep.

[26] Cedernaes J., Lampola L., Axelsson E.K., Liethof L., Hassanzadeh S., Yeganeh A. (2015). A single night of partial sleep loss impairs fasting insulin sensitivity but does not affect cephalic phase insulin release in young men. Journal of Sleep Research.

[27] Spaeth A.M., Dinges D.F., Goel N. (2013). Effects of experimental sleep restriction on weight gain, caloric intake, and meal timing in healthy adults. Sleep.

[28] Thaiss C.A., Zeevi D., Levy M., Zilberman-Schapira G., Suez J., Tengeler A.C. (2014). Transkingdom control of microbiota diurnal oscillations promotes metabolic homeostasis. Cell.

[29] Liang X., Bushman F.D., FitzGerald G.A. (2015). Rhythmicity of the intestinal microbiota is regulated by gender and the host circadian clock. Proceedings of the National Academy of Sciences of the United States of America.

[30] Zarrinpar A., Chaix A., Yooseph S., Panda S. (2014). Diet and feeding pattern affect the diurnal dynamics of the gut microbiome. Cell Metabolism.

[31] Cedernaes J., Sand F., Liethof L., Lampola L., Hassanzadeh S., Axelsson E.K. (2016). Learning and sleep-dependent consolidation of spatial and procedural memories are unaltered in young men under a fixed short sleep schedule. Neurobiology of Learning and Memory.

[32] Hildebrandt M.A., Hoffmann C., Sherrill-Mix S.A., Keilbaugh S.A., Hamady M., Chen Y.Y. (2009). High-fat diet determines the composition of the murine gut microbiome independently of obesity. Gastroenterology.

[33] WHO (2006). Definition and diagnosis of diabetes mellitus and intermediate hyperglycemia: report of a WHO/IDF consultation. http://www.who.int/diabetes/publications/Definition%20and%20diagnosis%20of%20diabetes_new.pdf.

[34] Cedernaes J., Osler M.E., Voisin S., Broman J.E., Vogel H., Dickson S.L. (2015). Acute sleep loss induces tissue-specific epigenetic and transcriptional alterations to circadian clock genes in men. Journal of Clinical Endocrinology Metabolism.

[35] Woting A., Pfeiffer N., Hanske L., Loh G., Klaus S., Blaut M. (2015). Alleviation of high fat diet-induced obesity by oligofructose in gnotobiotic mice is independent of presence of Bifidobacterium longum. Molecular Nutrition & Food Research.

[36] Silber M.H., Ancoli-Israel S., Bonnet M.H., Chokroverty S., Grigg-Damberger M.M., Hirshkowitz M. (2007). The visual scoring of sleep in adults. Journal of Clinical Sleep Medicine.

[37] Samuel B.S., Gordon J.I. (2006). A humanized gnotobiotic mouse model of host-archaeal-bacterial mutualism. Proceedings of the National Academy of Sciences of the United States of America.

[38] Samuel B.S., Hansen E.E., Manchester J.K., Coutinho P.M., Henrissat B., Fulton R. (2007). Genomic and metabolic adaptations of Methanobrevibacter smithii to the human gut. Proceedings of the National Academy of Sciences of the United States of America.

[39] Larsen N., Vogensen F.K., van den Berg F.W., Nielsen D.S., Andreasen A.S., Pedersen B.K. (2010). Gut microbiota in human adults with type 2 diabetes differs from non-diabetic adults. PLoS One.

[40] Ley R.E., Turnbaugh P.J., Klein S., Gordon J.I. (2006). Microbial ecology: human gut microbes associated with obesity. Nature.

[41] Benedict C., Hallschmid M., Lassen A., Mahnke C., Schultes B., Schioth H.B. (2011). Acute sleep deprivation reduces energy expenditure in healthy men. The American Journal of Clinical Nutrition.

[42] Matsuda M., DeFronzo R.A. (1999). Insulin sensitivity indices obtained from oral glucose tolerance testing: comparison with the euglycemic insulin clamp. Diabetes Care.

[43] Zhang H., DiBaise J.K., Zuccolo A., Kudrna D., Braidotti M., Yu Y. (2009). Human gut microbiota in obesity and after gastric bypass. Proceedings of the National Academy of Sciences of the United States of America.

[44] Spencer M.D., Hamp T.J., Reid R.W., Fischer L.M., Zeisel S.H., Fodor A.A. (2011). Association between composition of the human gastrointestinal microbiome and development of fatty liver with choline deficiency. Gastroenterology.

[45] Claus S.P., Ellero S.L., Berger B., Krause L., Bruttin A., Molina J. (2011). Colonization-induced host-gut microbial metabolic interaction. MBio.

[46] Ley R.E., Backhed F., Turnbaugh P., Lozupone C.A., Knight R.D., Gordon J.I. (2005). Obesity alters gut microbial ecology. Proceedings of the National Academy of Sciences of the United States of America.

[47] Everard A., Lazarevic V., Gaia N., Johansson M., Stahlman M., Backhed F. (2014). Microbiome of prebiotic-treated mice reveals novel targets involved in host response during obesity. ISME Journal.

[48] Poroyko V.A., Carreras A., Khalyfa A., Khalyfa A.A., Leone V., Peris E. (2016). Chronic sleep disruption alters gut microbiota, induces systemic and adipose tissue inflammation and insulin resistance in mice. Scientific Reports.

[49] Jumpertz R., Le D.S., Turnbaugh P.J., Trinidad C., Bogardus C., Gordon J.I. (2011). Energy-balance studies reveal associations between gut microbes, caloric load, and nutrient absorption in humans. The American Journal of Clinical Nutrition.

[50] Broussard J.L., Ehrmann D.A., Van Cauter E., Tasali E., Brady M.J. (2012). Impaired insulin signaling in human adipocytes after experimental sleep restriction: a randomized, crossover study. Annals of Internal Medicine.

[51] Rao M.N., Neylan T.C., Grunfeld C., Mulligan K., Schambelan M., Schwarz J.M. (2015). Sub-chronic sleep restriction causes tissue specific insulin resistance. Journal of Clinical Endocrinology Metabolism.

[52] Buxton O.M., Pavlova M., Reid E.W., Wang W., Simonson D.C., Adler G.K. (2010). Sleep restriction for 1 week reduces insulin sensitivity in healthy men. Diabetes.

[53] Martinez I., Perdicaro D.J., Brown A.W., Hammons S., Carden T.J., Carr T.P. (2013). Diet-induced alterations of host cholesterol metabolism are likely to affect the gut microbiota composition in hamsters. Applied and Environmental Microbiology.

[54] Martinez I., Wallace G., Zhang C., Legge R., Benson A.K., Carr T.P. (2009). Diet-induced metabolic improvements in a hamster model of hypercholesterolemia are strongly linked to alterations of the gut microbiota. Applied and Environmental Microbiology.

[55] Zhang C., Zhang M., Wang S., Han R., Cao Y., Hua W. (2010). Interactions between gut microbiota, host genetics and diet relevant to development of metabolic syndromes in mice. ISME Journal.

[56] Fleissner C.K., Huebel N., Abd El-Bary M.M., Loh G., Klaus S., Blaut M. (2010). Absence of intestinal microbiota does not protect mice from diet-induced obesity. British Journal of Nutrition.

[57] Cappuccio F.P., Taggart F.M., Kandala N.B., Currie A., Peile E., Stranges S. (2008). Meta-analysis of short sleep duration and obesity in children and adults. Sleep.

[58] Kaneita Y., Uchiyama M., Yoshiike N., Ohida T. (2008). Associations of usual sleep duration with serum lipid and lipoprotein levels. Sleep.

[59] Gangwisch J.E., Malaspina D., Babiss L.A., Opler M.G., Posner K., Shen S. (2010). Short sleep duration as a risk factor for hypercholesterolemia: analyses of the National Longitudinal Study of Adolescent Health. Sleep.

[60] Wolever T.M., Brighenti F., Royall D., Jenkins A.L., Jenkins D.J. (1989). Effect of rectal infusion of short chain fatty acids in human subjects. American Journal of Gastroenterology.

[61] Wolever T.M., Spadafora P., Eshuis H. (1991). Interaction between colonic acetate and propionate in humans. The American Journal of Clinical Nutrition.

[62] Veech R.L., Gitomer W.L., King M.T., Balaban R.S., Costa J.L., Eanes E.D. (1986). The effect of short chain fatty acid administration on hepatic glucose, phosphate, magnesium and calcium metabolism. Advances in Experimental Medicine and Biology.

[63] Wolever T.M., Spadafora P.J., Cunnane S.C., Pencharz P.B. (1995). Propionate inhibits incorporation of colonic [1,2-13C]acetate into plasma lipids in humans. The American Journal of Clinical Nutrition.

[64] Rahat-Rozenbloom S., Fernandes J., Gloor G.B., Wolever T.M. (2014). Evidence for greater production of colonic short-chain fatty acids in overweight than lean humans. International Journal of Obesity (London).

[65] Gao Z., Yin J., Zhang J., Ward R.E., Martin R.J., Lefevre M. (2009). Butyrate improves insulin sensitivity and increases energy expenditure in mice. Diabetes.

[66] Chambers E.S., Viardot A., Psichas A., Morrison D.J., Murphy K.G., Zac-Varghese S.E. (2015). Effects of targeted delivery of propionate to the human colon on appetite regulation, body weight maintenance and adiposity in overweight adults. Gut.

[67] Lin H.V., Frassetto A., Kowalik E.J., Nawrocki A.R., Lu M.M., Kosinski J.R. (2012). Butyrate and propionate protect against diet-induced obesity and regulate gut hormones via free fatty acid receptor 3-independent mechanisms. PLoS One.

[68] Summa K.C., Voigt R.M., Forsyth C.B., Shaikh M., Cavanaugh K., Tang Y. (2013). Disruption of the circadian clock in mice increases intestinal permeability and promotes alcohol-induced hepatic pathology and inflammation. PLoS One.

[69] Ferrell J.M., Chiang J.Y. (2015). Short-term circadian disruption impairs bile acid and lipid homeostasis in mice. Cellular and Molecular Gastroenterology and Hepatology.

[70] Ma K., Xiao R., Tseng H.T., Shan L., Fu L., Moore D.D. (2009). Circadian dysregulation disrupts bile acid homeostasis. PLoS One.

[71] Kupfer R.M., Northfield T.C. (1983). Diurnal variation in cholesterol saturation of gall-bladder bile. Gut.

[72] Nicholson J.K., Holmes E., Kinross J., Burcelin R., Gibson G., Jia W. (2012). Host-gut microbiota metabolic interactions. Science.

[73] Sonnenburg J.L., Backhed F. (2016). Diet-microbiota interactions as moderators of human metabolism. Nature.

[74] Le Chatelier E., Nielsen T., Qin J., Prifti E., Hildebrand F., Falony G. (2013). Richness of human gut microbiome correlates with metabolic markers. Nature.

[75] Serino M., Luche E., Gres S., Baylac A., Berge M., Cenac C. (2012). Metabolic adaptation to a high-fat diet is associated with a change in the gut microbiota. Gut.

[76] Broussard J.L., Wroblewski K., Kilkus J.M., Tasali E. (2016). Two nights of recovery sleep reverses the effects of short-term sleep restriction on diabetes risk. Diabetes Care.

[77] Leproult R., Deliens G., Gilson M., Peigneux P. (2015). Beneficial impact of sleep extension on fasting insulin sensitivity in adults with habitual sleep restriction. Sleep.

[78] Keller J., Groger G., Cherian L., Gunther B., Layer P. (2001). Circadian coupling between pancreatic secretion and intestinal motility in humans. American Journal of Physiology. Gastrointestinal and Liver Physiology.

[79] Sze M.A., Schloss P.D. (2016). Looking for a signal in the noise: revisiting obesity and the microbiome. MBio.

