# Supplementary material for: Gut microbiota and glucometabolic alterations in response to recurrent partial sleep deprivation in normal-weight young individuals
Source: Mol Metab. 2016 Oct 24;5(12):1175–86. doi: 10.1016/j.molmet.2016.10.003 (PMC5123208; doi:10.1016/j.molmet.2016.10.003)
Supplement: Supplementary file 1 [file mmc1.pdf]

Benedict et al. (Supplementary material)

Supplementary Figure 1. **(A)** Neither sleep condition nor fecal collection day (time effect) were found to be significant drivers for beta diversity using weighted abundances (baseline vs day 2; or day 2 samples for Sleep vs PSD). **(B)** Hierarchical clustering of samples across participant, BMI, cage timepoints and conditions (partial sleep deprivation, PSD, or normal sleep, NS) revealed clustering primarily according to participant. d0, day 0 (baseline sample); d2, day 2. Subjects have been arbitrarily numbered.

Fig 1A

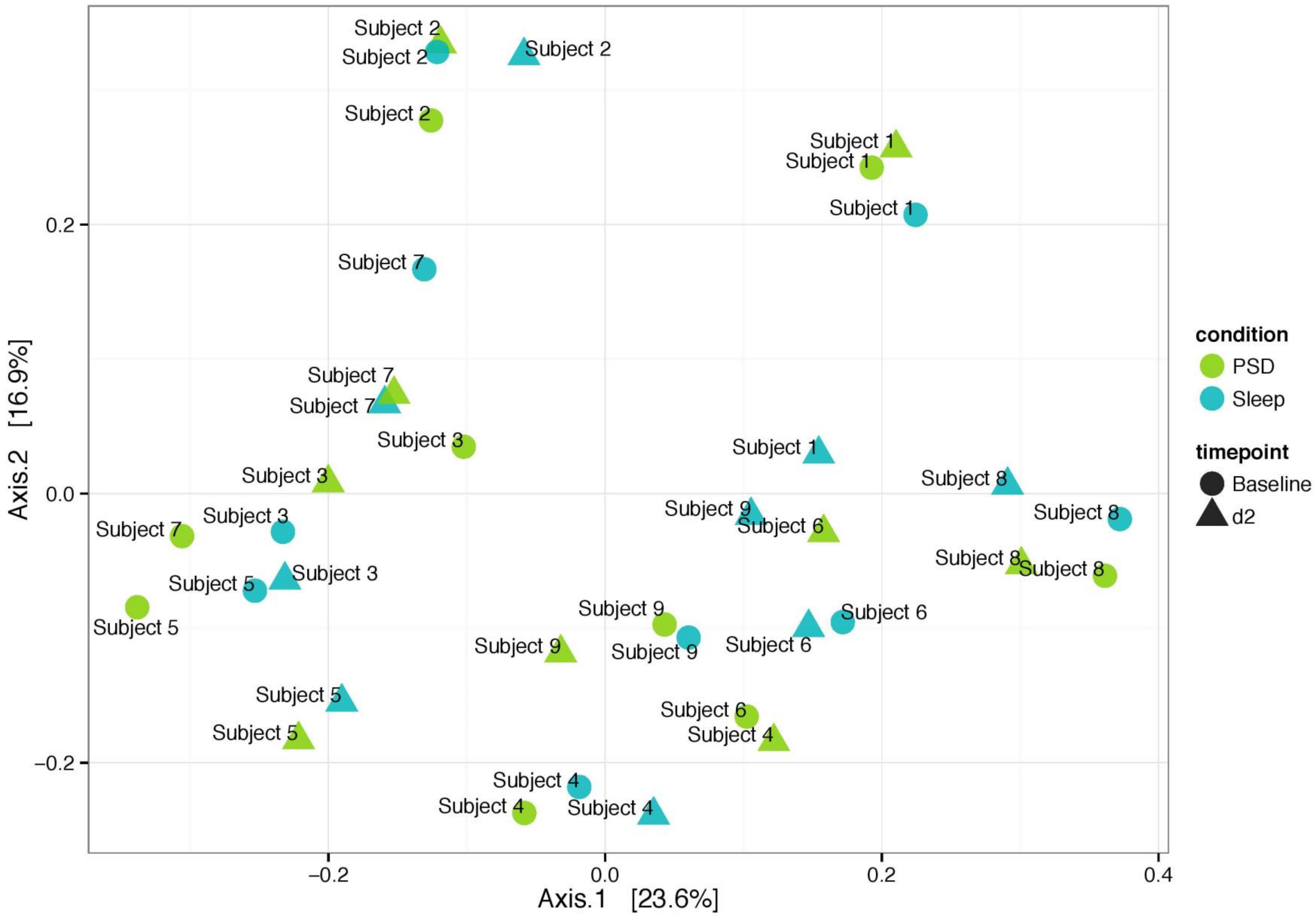

Subject 2
Subject 2
Subject 2
Subject 2
Subject 1
Subject 1
Subject 1
Subject 7
Subject 7
Subject 7
Subject 3
Subject 3
Subject 1
Subject 8
Subject 3
Subject 7
Subject 9
Subject 6
Subject 8
Subject 5
Subject 3
Subject 8
Subject 8
Subject 5
Subject 9
Subject 6
Subject 9
Subject 9
Subject 6
Subject 5
Subject 5
Subject 6
Subject 4
Subject 4
Subject 4
Subject 4
0.2
0.0
−0.2
Axis.2 [16.9%]
−0.2
0.0
0.2
0.4
Axis.1 [23.6%]
condition
PSD
Sleep
timepoint
Baseline
d2

Fig 1B

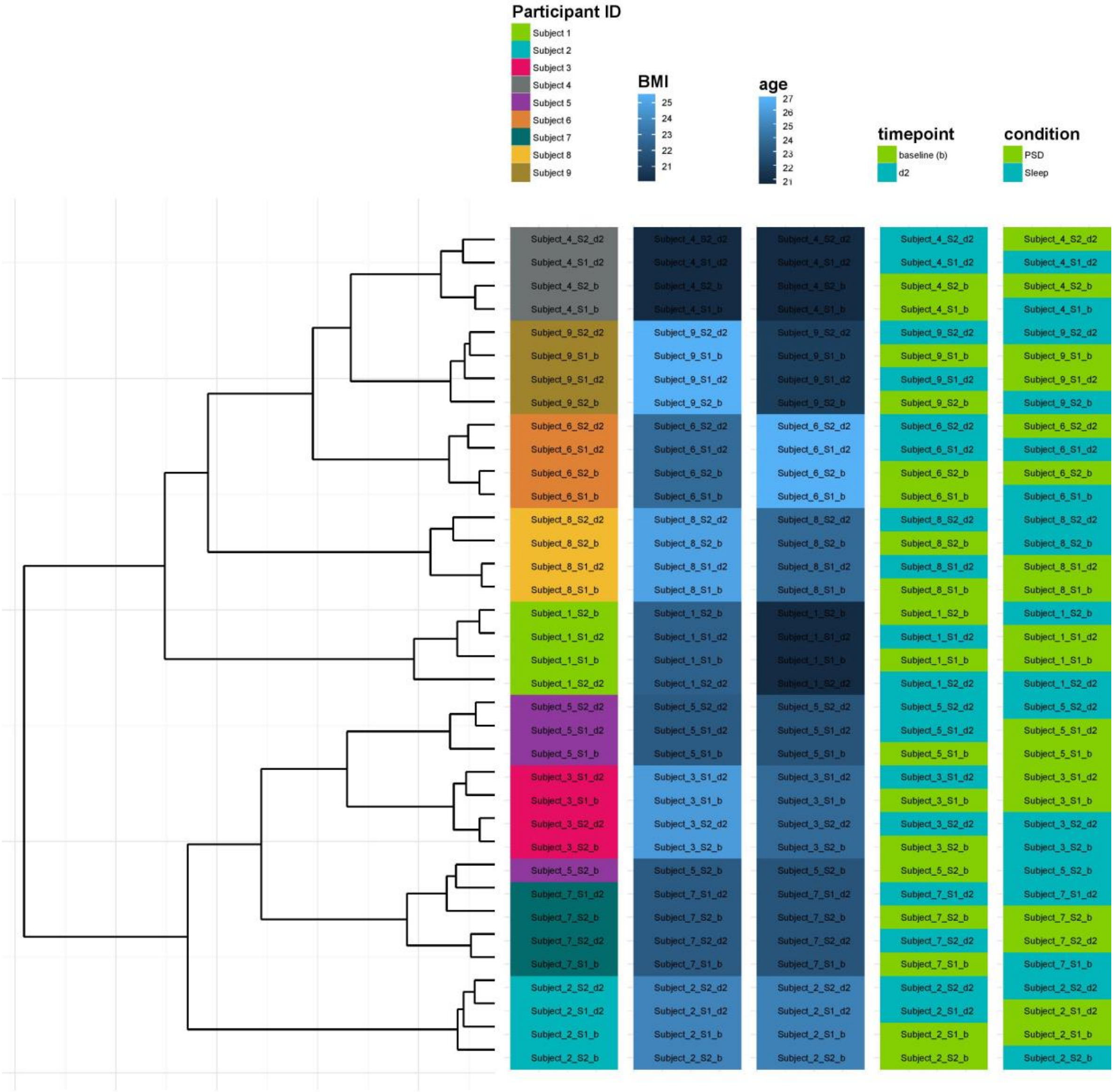

Participant ID
Subject 1
Subject 2
Subject 3
Subject 4
Subject 5
Subject 6
Subject 7
Subject 8
Subject 9
BMI
25
24
23
22
21
age
27
26
25
24
23
22
21
timepoint
baseline (b)
d2
condition
PSD
Sleep

Supplementary Figure 2. Bacterial composition at the (**A**) phylum and (**B**) family taxa level after two nights of partial sleep deprivation (PSD) or normal sleep ("Sleep").

Fig 2A

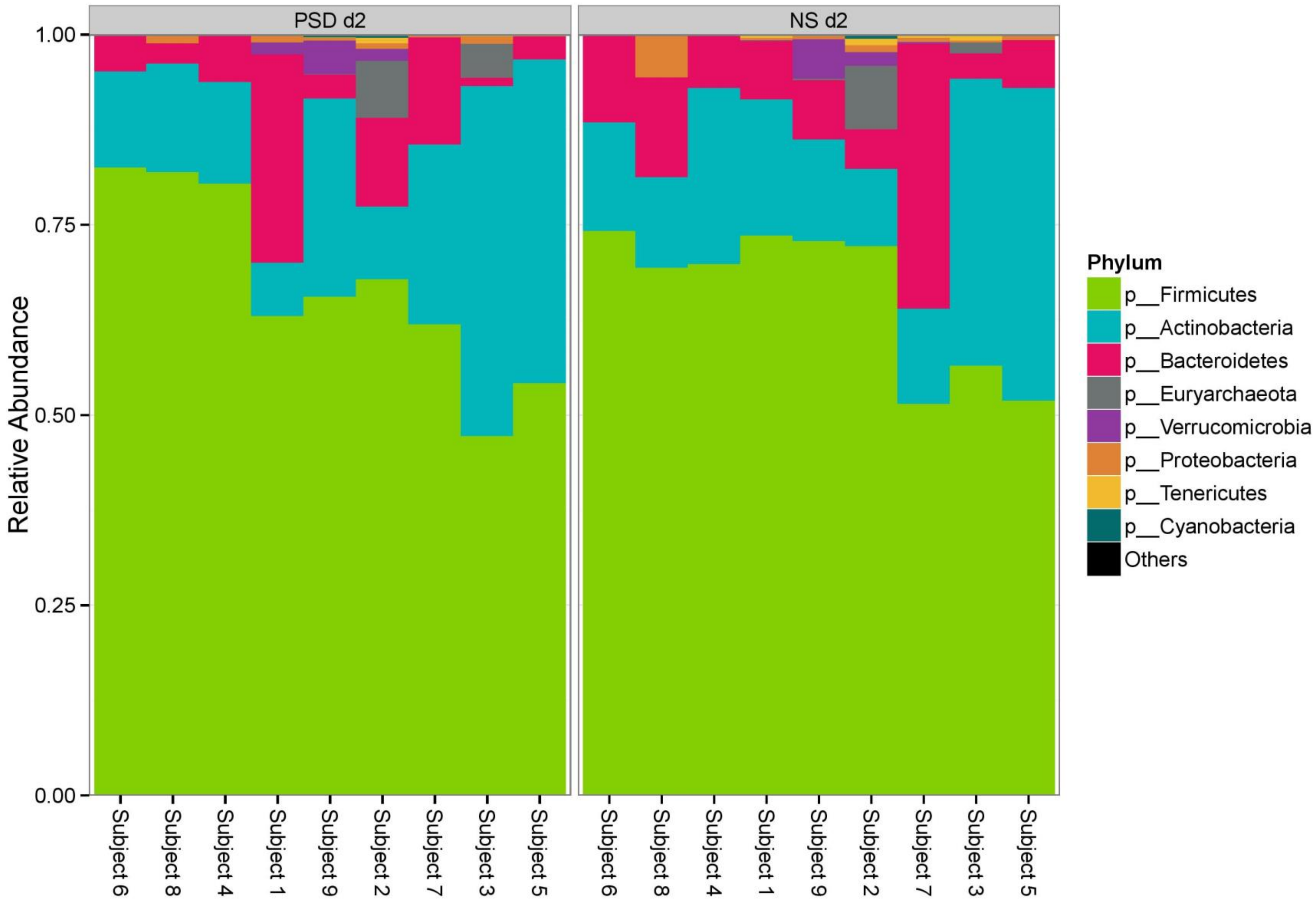

PSD d2
NS d2
Relative Abundance
1.00
0.75
0.50
0.25
0.00
Subject 6
Subject 8
Subject 4
Subject 1
Subject 9
Subject 2
Subject 7
Subject 3
Subject 5
Phylum
p__Firmicutes
p__Actinobacteria
p__Bacteroidetes
p__Euryarchaeota
p__Verrucomicrobia
p__Proteobacteria
p__Tenericutes
p__Cyanobacteria
Others

Fig 2B

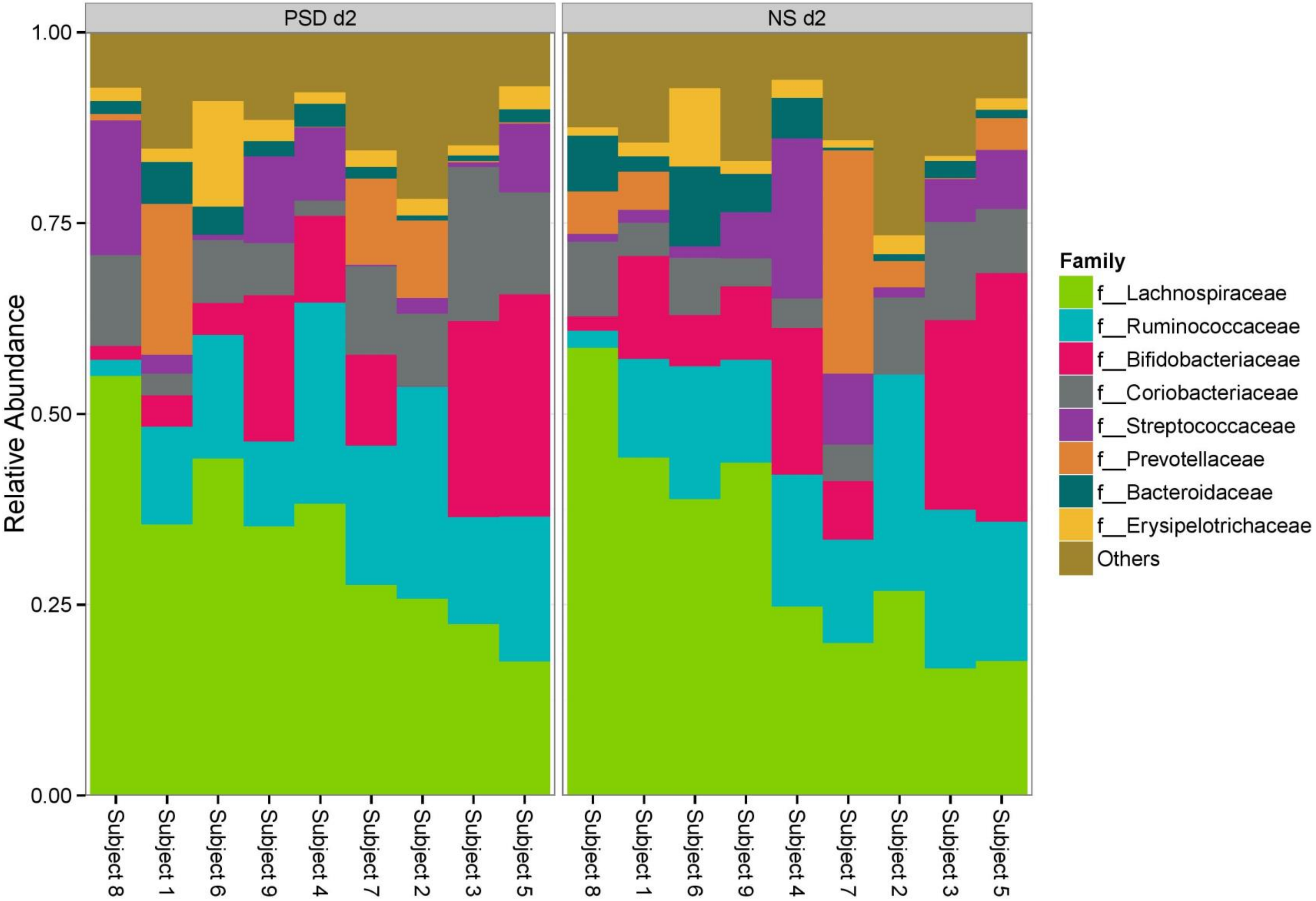

PSD d2
NS d2
1.00
0.75
0.50
0.25
0.00
Relative Abundance
Subject 8
Subject 1
Subject 6
Subject 9
Subject 4
Subject 7
Subject 2
Subject 3
Subject 5
Family
f__Lachnospiraceae
f__Ruminococcaceae
f__Bifidobacteriaceae
f__Coriobacteriaceae
f__Streptococcaceae
f__Prevotellaceae
f__Bacteroidaceae
f__Erysipelotrichaceae
Others

Supplementary Table 1. Number of categories observed at each taxonomic rank following sequencing of the feces samples obtained after normal sleep and partial sleep deprivation.

Rank	Number of Types
Kingdom	2
Phylum	14
Class	27
Order	45
Family	136
Genus	398
Species	546

Supplementary Table 2. Alpha diversity measures according to timepoint and condition, using both observed and Shannon's method. Tested with Kruskal-Wallis rank sum test with Dunn's post-hoc test, followed by Bonferroni correction for multiple comparisons.

**Formula**	**Value**	**Chi-square**	**P value**
~ *timepoint*	*Observed*	1.766	0.18
~ *condition*	*Observed*	0.324	0.57
~ *timepoint*	*Shannon*	0.677	0.41
~ *condition*	*Shannon*	0.009	0.92
